# Small-Cell Lung Cancer: 8 Years Experience of a Single Multidisciplinary Team

**DOI:** 10.1155/2008/150760

**Published:** 2008-10-16

**Authors:** Loaie M. El-Helw, Trevor K. Rogers, Matthew Q. F. Hatton

**Affiliations:** ^1^Chest Department and Lung Cancer Clinic, Doncaster and Bassetlaw Hospitals NHS Foundation Trust, Doncaster Royal Infirmary, Doncaster DN2 5LT, UK; ^2^Department of Clinical Oncology, Weston Park Hospital, Sheffield S10 2SJ, UK

## Abstract

*Aims*. We have audited the changes in treatment practice for small-cell lung cancer (SCLC) presented to a single multidisciplinary team (MDT) at Doncaster and Bassetlaw Hospitals between January 1998 and December 2005. *Materials and Methods*. The MDT database was used to identify all patients with SCLC. Anonymised demographic, treatment, and outcome details were extracted from the database supplemented by patient records. *Results*. 235 patients were identified. 112 (48%) had limited disease at presentation. Chemotherapy was the initial treatment for 195 patients, 77% of whom had a documented radiological response with a complete response in 24%. Chemotherapy regimes evolved during the study period with the increasing use of platinum-based chemotherapy. Anthracycline-based chemotherapy was most used before 2004 and was given to 57% of all patients. 42% received consolidation thoracic radiotherapy and 24% prophylactic cranial irradiation. The median and 2-year survival were 8 months and 18%, respectively, for patients with limited disease and 5 months and 5%, respectively, for extensive disease. *Conclusion*. We have documented changes in treatment practice and service delivery of SCLC over the 8 years during which the MDT has been operating. However, there has not achieve any significant improvement in outcome for the population of patients with SCLC.

## 1. Introduction

It has been recognised that cancer survival in the UK has lagged behind the USA and other European countries. This recognition triggered the Calman-Hine report in the mid 1990s, which started a major reorganisation of cancer services in the UK. In 1998, the NHS executive published guidance on Improving Outcomes in Lung Cancer [1]. One aspect of this guidance examined was the effectiveness of multidisciplinary teams (MDTs) and supported the recommendation that all patients with lung cancer have their case reviewed by a specialist MDT and targeted investment was made to develop team working. In 2000, the NHS cancer plan looked to introduce more radical changes and cover the whole cancer care pathway. This led to significant investment aimed at reducing waiting times and improving access to treatment.

The Doncaster lung cancer MDT was set up and in 1997 started to review the management of all patients with lung cancer who presented to three district general hospitals that cover a population of 450000 patients in South Yorkshire and North Nottinghamshire . From the outset, a database was designed to prospectively record data for all patients reviewed by the MDT and included demographic, staging, and treatment details. The MDT has now been operational for 10 years and we have started to review our experience over that time particularly looking to document the trends in treatment and outcome that have occurred during this period of cancer service reorganisation in the UK.

This paper has looked specifically at patients with small-cell lung cancer (SCLC) which compromises approximately 20% of all cases of lung cancer. The disease is characterised by early metastatic spread, sensitivity to chemotherapy, and early development of resistance [2]. Untreated the median survival in limited stage is 3 months, and in extensive stage 1.5 months. With single-agent chemotherapy, trials indicate a median survival of 6 and 4 months and with combination chemotherapy, 10–20 and 7–11 months, respectively [3–6].

Improving outcomes in lung cancer, published in 1998, [1] reviewed the research evidence and recommended the use of combination chemotherapy. This guidance was able to suggest an optimal duration of chemotherapy but concluded there was little clear evidence to guide the choice of drugs. The guidance also felt there was sufficient evidence to support the use of thoracic radiotherapy and prophylactic cranial irradiation in limited stage disease. The National Institute for Clinical Excellence commissioned an update, which was published as the “The Diagnosis and Treatment of Lung Cancer Guidelines” in 2005 [7]. This guidance built on the previous recommendation indicating the superiority of platinum-based chemotherapy as first line treatment and indicating a role for second line treatment in selected patients. No new recommendations were made for radiotherapy, though its timing and sequencing with chemotherapy was discussed.

Therefore, we have chosen to study the whole population of patients with SCLC presenting to our MDT and audit the changes in treatment practice that have occurred between 1998 and 2005 and monitored the effect that the investment made following the NHS cancer plan has had an outcome.

## 2. Materials and Methods

The MDT database has prospectively recorded all patients with a histological or radiological diagnosis of lung cancer since the Doncaster Lung cancer MDT was formed. This database has been used to identify all patients with a confirmed histological diagnosis of SCLC between January 1998 and December 2005. The demographic and treatment data on the database, supplemented by information from the patient records, has been anonymised and subjected to statistical analysis. To examine for time trends we divided patients into 3 chronological groups (1998–2000, 2001-2002, and 2003–2005) each containing approximately 80 patients. Kaplan Myer survival analysis was performed using SPSS statistical package version 12.0.1.

### 2.1. Patients Characteristics

Two hundreds and thirty five patients with SCLC were identified; 120 (51%) females and 115 (49%) males. The median age was 66 years (range 25–87), there were 83 patients (35%) ≥70 years old at the time of diagnosis. A histological diagnosis was made at flexible bronchoscopy in 83%, the remaining 17% required fine needle aspiration for cytology or CT guided biopsy.

110 patients had a performance status of 0 or 1 at diagnosis and staging investigations showed 112 patients had limited stage disease confined to the ipislateral lung and mediastinum. Applying the Manchester prognostic scoring system [8], 91 patients (39%) fell into the good prognosis category, 122 (52%) the intermediate, and 22 (9%) were in the poor prognosis group.

**Figure 1:**
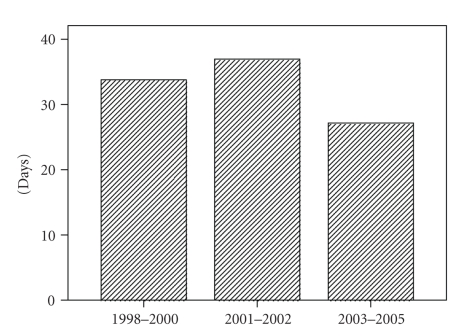
Mean time from referral to treatment.

We analysed the time from GP referral to starting definitive treatment as a measure of the early diagnostic part of the patient pathway. The duration was shortest between 2003–2005 compared to those of 2001-2002 and 1998–2000 (mean 27, 37, and 34 days and median of 26, 33, and 31 days, resp.) (*P* < .001 using one way ANOVA test) (Figure 1).

In Table 1, the patient characteristics and treatment outcome are summarised and broken down into the three chronological groups. For 83% of patients the initial treatment was chemotherapy. The table also shows that anthracycline-based chemotherapy was most commonly used overall, though over the study period there was increasing use of platinum-based treatment, which became the most commonly used treatment in the last three years of the study. The median number of chemotherapy cycles given was 6 (range 1–6) and seventeen patients received chemotherapy as part of ongoing clinical trials.

The table also indicates that the use of radiotherapy was constant over the study period. Around 5% of patients received radiotherapy as initial palliative treatment and in a further 18% it was used to palliate symptoms later in the course of the disease. Consolidation thoracic radiotherapy (TRT) was given in 42% of patients who had shown a response to the initial chemotherapy and prophylactic cranial irradiation (PCI) was given to 24% of patients who presented with limited stage disease and responded to chemotherapy or were treated in the trial setting.

## 3. Results

### 3.1. Response

A Total 196 patient received primary chemotherapy, 5 died following the first cycle of treatment leaving 191 assessable for response (Table 2). There was an overall response rate was 77% (24% CR and 53% PR) with only 6% having documented progressed during chemotherapy. We found a higher response rate for patients with limited disease, performance status 0/1, and those less than 70 years old but none reached statistical significance (using chi-square test, *P*-values were 0.090, 0.390, 0.702, resp.). There was higher response rate with platinum-based treatments (87%) compared anthracycline (78%). The high response rate for platinum-based treatment includes patients in the poor prognostic group, particularly those with impaired hepatic function, in whom single-agent carboplatin was used and had a response rate of 50%.

### 3.2. Survival

**Figure 2:**
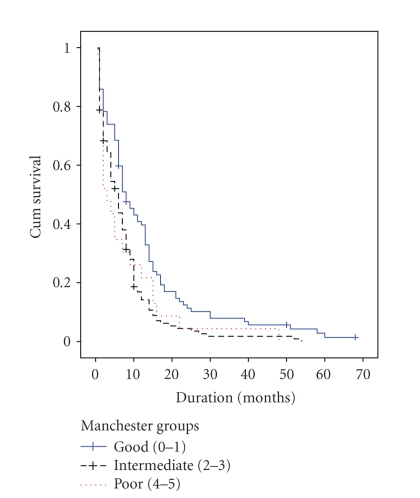
Survival in relation to Manchester scores.

**Figure 3:**
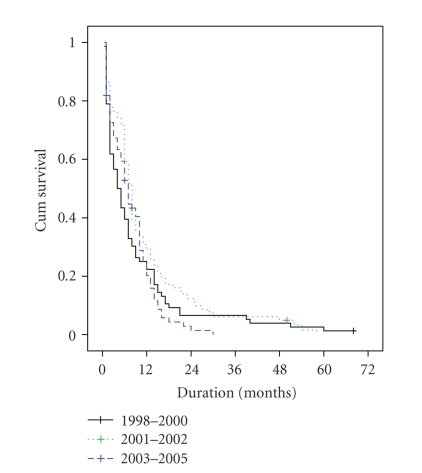
Survival function in relation to chronological periods.

The overall median survival, and 2- and 5-year survival rates for all patients were 6 months, 8%, and 2%, respectively. In limited stage disease, survivals were 8 months, 15%, and 5%, respectively, and in extensive stage disease 5 months, 5%, and 0%, respectively for (*P* < .0001). Univariate analysis also showed good Manchester prognostic group (Figure 2) and chemotherapy response to be associated with improved survival, but no significant survival differences were seen with age, sex, and year of treatment (Figure 3).

### 3.3. Toxicity

Five patients (2.5%) died in the 3 weeks that followed the first cycle of chemotherapy, and were recorded as treatment related deaths. Case-notes review indicated 14% were admitted for the treatment of neutropenic sepsis episodes, with this complication being more common with anthracycline-based treatment (10%) than platinum (4%).

### 3.4. Relapse

183 patients (96%) relapsed after primary chemotherapy treatment, mostly with metastatic recurrence (70%), 40% of the patients received second line chemotherapy which included CAV regimen, single-agent carboplatin or carboplatin/etoposide +/− palliative radiotherapy. The median survival following second line treatment was 2 months (range 1–12 months).

## 4. Discussion

The Doncaster MDT serves an area with significantly higher rates of lung cancer incidence and mortality than the national average; the indirectly standardised registration rate (SRR) (2001–2003) for Doncaster was 138 for men and 140 for women [9]. Overall the standardised mortality ratios (SMR) in Doncaster area were 114, 112 for males and females, respectively, which are among the highest SMR in England and Wales [10]. This indicates a poor general health status for the population in the Doncaster area, which would be expected to have an effect on outcome following a diagnosis of small-cell lung cancer in various ways. Our population of patients with SCLC will be different to the population of patients entering the trials that provides our evidence base. An example would be a comparison of our demographics with the Norwegian Lung Cancer Study Group Trial [11], which reported in the middle of our study period and has relatively wide entry criteria that included limited and extensive disease. The age, performance status, and stage of our patients are similar to those included in the trial but we note we treated a significantly higher proportion of female patients (51 vs. 36%) with worse performance status (PS 0/1 47% vs. 65%).

The treatment response rates documented in this study are comparable to those reported in trials, but these trials [12] have reported better survival outcomes. The Norwegian study [11], for example, compared anthracycline and platinum-based chemotherapy and demonstrated a survival advantage in favour of the platinum-based treatment (median survival 7.8 vs. 10.2) months. Although the entry criteria for this study were relatively broad, our population included a number of patients who would not meet the trial inclusion criteria, the biggest group being the 17% of our patients who were not considered fit for primary chemotherapy treatment.

The national LUCADA database has been developed to collect more detailed demographic information on patients with lung cancer, to allow adjustments for comorbidity and other factors to be made when comparing outcome across England and Wales and has published its first report [13] with coverage extending to the majority of the population. The data collected by this report covers a different, but overlapping, period to our audit and comparison suggests the median survival reported in our series is a typical outcome for UK as a whole (6 vs. 5.6 months), using current guidelines. During the period of our audit, our unit reached the current national guidance recommendations with 100% of patients being reviewed by the MDT and histological confirmation rate of 83% and this compares favourable with the LUCADA averages in 2005 of 78% and 65%, respectively. In addition, treatment rates compare favourably, 83% of our patients received chemotherapy; the LUCADA average being 54%. Therefore, with median age, sex ratio, performance status, stage of disease, and comorbidities being similar, we are disappointed that at best we demonstrate a marginal improvement in the median survival of our patients with SCLC (6 months) compared to LUCADA report (5.6 months).

Over the 8-year study period there were no significant demographic changes, though we did notice a trend towards a higher female incidence and extensive stage disease at initial presentation, the latter probably reflecting increased accuracy of staging with the increased use of CT scanning. During the audit period there was accumulating evidence indicating the superiority of platinum-based treatment over anthracyclines [11, 14, 15]. This was reflected in the guidelines published in 2005 [7] and our practice with a clear trend towards the increased use of platinum treatment in the third cohort of patients. There was no change in the guideline advice on the use of radiotherapy over the study period as the evidence demonstrating the benefit of thoracic radiotherapy [16], and prophylactic cranial irradiation [17] had accumulated during the 1990s and was being applied at the start of the audit period. There was no change in the proportion of patients receiving radiotherapy over time, though from 2003 we did participate in a study of PCI in extensive stage patients and considered concurrent rather than sequential thoracic radiotherapy [18, 19].

The changes in treatment practice that have occurred over the 8 years have been small; so perhaps it is unsurprising that they have not fed through to any measurable effect on outcome for the total population with small-cell lung cancer. However, the bigger changes that occurred during our audit period were driven by the NHS cancer plan, which revolutionised the delivery of cancer treatment across the UK. The plan focused attention on target times for access, diagnosis, and waiting times, and significant investment was made to reduce the intervals between referral, first hospital appointment and treatment. These targets are now being met for 96–99% of cancer patients and this audit was able to document an improvement in the patient pathway. Comparing in 1998–2000 with 2003–2005 there was a reduction in the mean time from GP referral to a management plan being agreed (from 31 to 18 days) and in the mean time from referral to initial treatment (from 34 days 27 days). The disappointment is that we are unable to document any significant improvement in outcome to correspond with this improvement.

## 5. Conclusion

We feel that these data reflect the evolution of evidence-based treatment, delivered to an unselected cohort of patients presenting to a single cancer unit. Disappointingly, we could not document any significant improvement in outcome for patients over the 8-year audit period. It remains difficult to translate the survival benefits reported in trials. We feel that improvements in outcome will only come with earlier diagnosis and improvements in the general health of our population.

## Figures and Tables

**Table 1:** Patients characteristics and treatment outcome over the study period.

Variables	Number of patients (%)			Total no. (%)
Years (inclusive)	January 1998 till December 2000	January 2001 till December 2002	January 2003 till December 2005	
*Patients number *	76	82	77	235
*Gender *				
Females	35 (46)	44 (54)	41 (53)	120 (51)
Males	41 (54)	38 (46)	36 (47)	115 (49)
*Stage*				
LD	39 (51)	38 (46)	35 (45)	112 (48)
ED	37 (49)	44 (54)	42 (55)	123 (52)
*Mean time from referral to starting treatment (days)*	34	37	27	Overall 31
*Treatment *				
Chemotherapy	63 (83)	68 (83)	64 (83)	195 (83)
Palliative RT	4 (5)	5 (6)	4 (5)	13 (5.5)
Supportive Care	8 (11)	9 (11)	9 (12)	26 (11)
Resection and chemotherapy	1 (1)	—	—	1 (0.5)
*Chemoregimens*				
Anthracycline based	38 (59)	43 (63)	30 (47)	111 (57)
Platinum based	14 (22)	25 (37)	34 (53)	73 (37)
Others	12 (19)	—	—	12 (6)
*Radiotherapy *				
PCI	19 (33)	20 (35)	18 (32)	57 (24)
Consolidation TRT	32 (32.4)	33 (33.3)	34 (34.3)	99 (42)
To Metastases	9 (21)	22 (51)	12 (28)	43 (18)
*Median survival *				
For all patients (m)	4	8	7	6 (*P* = .143)
For patients with LD (m)	7	8	10	8 (*P* = .516)
For patients with ED (m)	3	6	5	5 (*P* = .006)

*P*-values were calculated using logrank test				

LD: limited disease, ED: extensive disease, RT: radiotherapy, PCI: prophylactic cranial irradiation, TRT: thoracic radiotherapy, m: months.

**Table 2:** Response to chemotherapy among 191 assessable patients.

		Response, no. (%)			Total
	CR	PR	SD	PD	
*Variable *					
*Overall response *	46 (24)	101 (53)	32 (17)	12 (7)	191
*Stage *					
Limited	25 (27)	52 (58)	9 (10)	5 (5)	91
Extended	21 (21)	49 (49)	23 (23)	7 (7)	100
*Manchester groups*					
Good	27 (33)	44 (55)	6 (7)	4 (5)	81 (42)
Intermediate	19 (20)	46 (49)	22 (23)	8 (8)	95 (50)
Poor	—	11 (73)	4 (27)	—	15 (8)
*Age *					
<70 years	35 (27)	68 (51)	21 (16)	8 (6)	132 (69)
≥70 years	11 (19)	33 (56)	11 (19)	4 (6)	59 (31)
*Gender *					
Males	17 (17)	54 (55)	20 (20)	7 (8)	98 (51)
Females	29 (30)	47 (48)	12 (12)	5 (5)	93 (49)
*Chemotherapy *					
Anthracyclines-based	30 (28)	54 (50)	19 (18)	5 (4)	108 (57)
Platinum-based regimens	14 (30)	27 (57)	5 (11)	1 (2)	47 (25)
Carboplatin	2 (8)	10 (42)	6 (25)	6 (25)	24 (12)
Others	—				12 (6)

CR: complete response, PR: partial response, SD: stable disease, PD: progressive disease.
